# Ginger extract modulates the expression of IL-12 and TGF-β in the central nervous system and serum of mice with experimental autoimmune encephalomyelitis

**Published:** 2017

**Authors:** Abdollah Jafarzadeh, Reyhane Ahangar-Parvin, Maryam Nemat, Zahra Taghipour, Ali Shamsizadeh, Fatemeh Ayoobi, Zuhair Mohammad Hassan

**Affiliations:** 1 *Department of Immunology, Medical School, Rafsanjan University of Medical Sciences, Rafsanjan, Iran*; 2 *Department of Immunology, Medical School, Kerman University of Medical Sciences, Kerman, Iran*; 3 *Neuroscience Research Center, Institute of Neuropharmacology, Kerman University of Medical Sciences, Kerman, Iran*; 4 *Department of Histology, Medical School, Rafsanjan University of Medical Sciences, Rafsanjan, Iran*; 5 *Department of Physiology, Medical School, Rafsanjan University of Medical Sciences, Rafsanjan, Iran*; 6 *Department of Immunology, Medical School, Tarbiat Moddares University, Tehran, Iran*

**Keywords:** Experimental autoimmune encephalomyelitis, Ginger, IL-12, TGF-β, Serum

## Abstract

**Objective:**

The main function of IL-12 is differentiation of naive T cells intoTh1 cells and TGF-β is a powerful immunoregulatory cytokine. The immunomodulatory and anti-inflammatory properties of ginger have also been reported in some studies. The aim of this study was to evaluate the effects of ginger extract on the expression of IL-12 and TGF-β in a model of experimental autoimmune encephalomyelitis (EAE).

**Materials and Methods:**

EAE was induced in C57BL/6 mice by immunization with myelin oligodendroglial glycoprotein emulsified in complete Freund's adjuvant. The mice were administered intra-peritoneally with ginger extracts or PBS, from day +3 to +30. On day 31, mice were scarified and the expression of IL-12 and TGF-β mRNA in the spinal cord were determined by using real time-PCR. The serum levels of cytokines were measured by ELISA.

**Results:**

In PBS-treated EAE mice, the expression of IL-12 P35 and IL-12 P40 mRNA in the CNS and the mean serum levels of IL-12 were significantly higher than those of healthy group (p<0.001). In ginger-treated EAE mice, the expression of IL-12 mRNA and its serum levels were significantly lower as compared to PBS-treated EAE mice. No significant difference was observed between PBS-treated EAE mice and healthy group regarding the expression of TGF-β mRNA. In ginger (300 mg/kg)-treated EAE group, the expression of TGF-β mRNA and its serum levels were significantly higher in comparison to PBS-treated EAE mice (p<0.01 and p<0.05, respectively).

**Conclusion:**

These results indicated that ginger extract modulates the expression of IL-12 and TGF-β in CNS and serum of EAE mice.

## Introduction

Multiple sclerosis (MS) is a chronic neuroinflammatory disease which occurs as a result of demyelination of the central nervous system (CNS) (Milo et al., 2014 ▶). Experimental autoimmune encephalomyelitis (EAE) is an experimental model of human MS that is inducible in vulnerable animals by active immunization with myelin-derived antigens mixed with adjuvant (Robinson et al., 2014 ▶). T-helper (Th) cells-dependent immune responses, in particular, exert an important role in the pathogenesis of both MS and EAE diseases (Raphael et al., 2015 ▶). Upon antigenic stimulation, naïve Th cells differentiate into several subsets such as Th1, Th2, Th17 and regulatory T (Treg) cells which secrete distinct cytokines (Raphael et al., 2015 ▶). It has been reported that the autoreactive Th1 and Th17 cells were responsible for demyelination in MS and EAE (Buc, 2013 ▶; Raphael et al., 2015 ▶), whereas Th2 and Treg cells confer protection against these diseases (Buc, 2013 ▶). Our recent studies showed a higher levels of a Th17 cells-related chemokine (CCL20) and lower levels of a Th2/Treg cells-related chemokine (CCL22) in patients with MS disease (Jafarzadeh et al., 2014a ▶; Jafarzadeh et al., 2014b ▶). 

Structurally, IL-12, consisting of two covalently linked P35 and P40 subunits, is produced by activated antigen-presenting cells such as dendritic cells and macrophages (Lasek et al., 2014 ▶). IL-12 receptor is expressed on a number of immune cells, including NK cells, T and B lymphocytes (de Paus et al., 2013 ▶; Lasek et al., 2014 ▶). The main actions of IL-12 are increasing the production of IFN-γ from NK and T cells, stimulation of NK cells and CD8+ T cells, inducing the differentiation of CD4+ Th0 cells toward the Th1 cells, increasing antibody-dependent cellular cytotoxicity (ADCC) against tumor cells, inducing IgG synthesis and suppressing IgE production by B cells (Croxford et al., 2014 ▶; Lasek et al., 2014 ▶).

TGF-β is an important multifunctional cytokine with strong immunoregulatory effects which is produced mainly by Treg cells (Yoshimura et al., 2011 ▶). The diverse effects of TGF-β include down-regulation of IL-1, IL-12, TNF-α and TNF-β production, inducing the Treg cells differentiation, modulating TLR4 expression, inducing IL-10 production by macrophages, increasing the generation of tolerogenic DC, down-regulationof T cell responses to IL-12, modulating macrophage and microglia activation, down-regulation of cytokine-enhanced MHC class II expression and suppression of both Th1- and Th2 cells differentiation (Mirshafiey et al., 2009 ▶; Mantel et al., 2011 ▶). 

Defects in TGF-β lead to serious autoimmune disease, whereas its over-expression protects mice against autoimmune diseases (Mantel et al., 2011 ▶). There are a number of studies showing that TGF-β protects against EAE and MS. TGF-β prevents sensitized T cells from entering into the CNS (Mirshafiey et al., 2009 ▶). Treatment of EAE mice with anti-TGF-β antibody exacerbates EAE severity, whereas treatment with TGF-β suppresses the disease (Mantel et al., 2011 ▶). 

The rhizomes of the ginger (*Zingiber officinale*) are commonly used as a flavor or food supplement. There are some antioxidants and anti-inflammatory components in ginger rhizomes (Ahui et al., 2008 ▶; Haniadka et al., 2013 ▶). Some patients use ginger powder to attenuate the inflammatory swelling and pain of osteoarthritis or rheumatoid arthritis (Al-Nahain et al., 2014 ▶). The anti-inflammatory effects of ginger and its components have also been demonstrated in patients with type 2 diabetes (Priya Rani et al., 2011 ▶), osteoarthritis (Drozdov et al., 2012 ▶), rheumatoid arthritis (Ramadan et al., 2013 ▶; Al-Nahain et al., 2014 ▶), acute respiratory distress syndrome (Vahdat Shariatpanahi et al., 2013 ▶) and primary dysmenorrheal (Rahnama et al., 2012 ▶). The anti-inflammatory activities of ginger extract or its pungent constituents such as gingerol and shogaol have been also demonstrated in experimental animal models such as models of airway inflammation (Kuo et al., 2011 ▶), ulcerative colitis (Ajayi et al., 2015 ▶), neuro-inflammation (Ha et al., 2012 ▶) and gastric ulcers (Wang et al., 2011 ▶). 

We have recently reported that ginger-treated EAE mice exhibited mild signs of EAE, a delay in disease onset and low infiltration of the inflammatory cell into the CNS. Moreover, treatment with ginger extract modulates the expression of IL-27 and IL-33 mRNA in EAE mice (Jafarzadeh et al., 2014c ▶). The aim of this study was to investigate the effects of ginger extract on the expression of IL-12 and TGF-β in the CNS and serum of C57BL/6 mice with EAE that was induced by immunization with myelin oligodendroglial glycoprotein (MOG). 

## Materials and Methods

**Preparation of the ginger extract**

Ginger extract was prepared as previously explained (Jafarzadeh et al., 2014c). Briefly, *Zingiber officinale* (ginger) was purchased from a herbal institute in Isfahan, Iran. Verification of the plant was performed by a botany specialist and was recognized by Voucher Number: 86.1133.1. The hydro-alcoholic extract of ginger was prepared by maceration method. Here, 3 kg of fresh ginger rhizome was cut into small pieces, air dried and ground into a fine powder using a pestle and mortar. The ginger powder was hold in a suitable container and 2000 ml ethanol 50% was added and the mixture was left at room temperature for 15 hr. Subsequently, the solid parts was removed by filtration and combined extracts were concentrated at 40°C, so that the solvent was evaporated using a rotary evaporator to give an extract that was designated as an alcoholic extract. Finally, a semi-dried extract was obtained and then, the appropriate amount of the originated extract was calculated and dissolved into the proportional volume of PBS. The prepared extract was kept in a fridge until use.

**Mice**

Female (6-8 week old) C57BL/6 mice (Pasteur Institute, Tehran, Iran) were used during this study. Mice were maintained in a temperature-controlled environment with a 12hr light/12hr dark cycle and were administered with standard laboratory food and water *ad libitum*. All mice were housed in a room where the testing procedure was performed so as to minimize any stress response potentially induced by novel environmental cues. All experiments were conducted on-site at Kerman University of Medical Sciences and were performed in accordance with the recommendations of the Medical School Ethics Committee on Animal Experimentation. Protocols were also in accordance with the National Research Council Guide (NRC, 2011) and European Community Council Directive of 24 November 1986 (86/609/EEC) for the care of laboratory animals.

**Induction and scoring of EAE**

The EAE was induced by using a MOG peptide as previously explained (Jafarzadeh et al., 2014c). The MOG is a component of the myelin sheath of nerves in the CNS and is known as an important target auto-antigen in MS disease. Immunization of C57BL/6 mice with MOG35-55 peptide induces EAE in 100% of animals (Takeuchi C et al., 2013 ▶; Robinson et al., 2014 ▶). According to manufacturer guideline, the MOG35-55 peptide amino acid sequence was Met-Glu-Val-Gly-Trp-Tyr-Arg-Ser-Pro-Phe-Ser-Arg-Val-Val-His-Leu-Tyr-Arg-Asn-Gly-Lys. 

Briefly, C57BL/6 mice were injected subcutaneously (s.c) on day 0 with 400 μg of MOG35–55 peptide (Alexis, Switzerland) emulsified in complete Freund's adjuvant containing 5 mg/ml of *Mycobacterium tuberculosis* at two sites in the flank. The mice received two additional intra-peritoneal (i.p) injections of 250 ng of pertussis toxin on days 0 and 48 hr post immunization. Mice were weighed and evaluated daily for clinical symptoms of disease. The disease was scored based on the following criteria: 0 asymptomatic,1 loss of tail tone,2 flaccid tail,3 paralysis of one hind limb,4 paralysis of two hind limbs,5 forelimb and hind limb paralysis and 6 dead (Takeuchi et al., 2013 ▶). Paralyzed mice had free access to food and water.

**Planning of research**

Mice were classified into 4 groups (5-6 mice in each) as follows: Group I (healthy control group): Mice in this group were considered as healthy normal without EAE and only treated with PBS as vehicle. Group II (EAE negative control group): Mice in this group were considered as PBS-treated EAE group without receiving ginger extract. Group III (ginger-treated EAE group): The mice with EAE enrolled into this group and received 200 mg/kg ginger extract. Group IV (ginger-treated EAE group): The mice with EAE enrolled into this group and received 300 mg/kg ginger extract. 

The mice were immunized on day 0 by administration an emulsion of MOG peptide and complete Freund adjuvant containing *M. tuberculosis* to induce EAE. The mice were intra-peritoneally (i.p) administered with either vehicle (PBS) in control groups or ginger extract (200 or 300 mg/kg BW, every other day) from day +3 to +30 in treatment groups. The EAE clinical scores and body weight were evaluated until day 30. On day 31, all mice were scarified, the blood samples were collected and the spinal cords and brains were removed for more analyses.

**Real-time PCR**

The expression of IL-12 mRNA and TGF-β mRNA in the spinal cord was determined by RT-PCR. Total RNA was extracted from the spinal cord using Trizol Reagent (Invitrogen, Carlsbad, CA). The purity of the extracted RNA was determined by electrophoresis on an ethidium bromide pre-treated agarose gel along with measuring absorption by spectrophotometer and calculation of 260/280 ratio. The extracted RNA was converted to cDNA using a cDNA synthesis kit (Bionner, Korea) with both oligo (dT) and random hexamer primers. The process of reverse transcription was performed by the following protocol: 70°C for 10 min (without reverse transcription enzyme), 20°C for 1 min (cooling step), addition of reverse transcription enzyme, 42°C for 60 min, and the protocol was completed following the final step at 95°C for 10 min to terminate the activation of the reverse transcription enzyme. 

Real-time PCR was performed using a SYBR green master mix (Bionner, Korea), combined with 200 ng of template cDNA with the appropriate primers (Table 1) in a Bio-Rad CFX96 system (Bio-Rad Company, USA) using the following program: 1 cycle of 95°C for 15 min, 40 cycles of 95°C for 30 sec and 60°C for 30 sec and finally 72°C for 30 sec. Primers were synthesized by the Bionner Company (Korea). Real-time PCR was carried out in triplicate and the β-actin gene was applied as a housekeeping gene for normalization of the amplified signals of the target genes. The sequences of the used primers are demonstrated in Table 1. The quantity of cytokines mRNA in the spinal cord are expressed as units relative to the amount of β-actin mRNA. The dissociation stages, melting curves and quantitative analyses of the data were performed using CFX manager software version 1.1.308.111 (Bio-Rad, USA).

**Table 1:** The primers used for analysis of gene expression of IL-12 and TGF-β in the spinal cord

**Gene**	**Primer**
**IL-12 (P35) **	Forward: CCACCCTTGCCCTCCTAAAC Reverse: GTTTTTCTCTGGCCGTCTTCA
**IL-12 (P40) **	Forward:GGAAGCACGGCAGCAGAATA Reverse: AACTTGAGGGAGAAGTAGGAATGG
**TGF-β **	Forward: ATTCCTGGCGTTACCTTG Reverse: GTATTCCGTCTCCTTGGTTC
**β-Actin **	Forward: AGAGGGAAATCGTGCGTGAC Reverse: CAATAGTGATGACCTGGCCGT

**Detection of serum levels of IL-12 and TGF-β**

On day 31, blood samples were collected via cardiac puncture and the sera were stored at -20°C. Serum levels of IL-12 and TGF-β were measured using commercial enzyme-linked immunosorbent assay (ELISA) kits (eBioscience, UK). The levels of sensitivity of the IL-12 and TGF-β kits were 4.0 and 15.0 pg/ml, respectively.

**Statistical analysis**

Data are presented as mean ± SEM. Statistical significance was determined by using ANOVA or Student's t test, if necessary. The p values of less than 0.05 were considered statistically significant.

## Results

**The effect of ginger extract on the clinical and pathological symptoms of EAE**

The effects of ginger extract on the clinical and pathological symptoms were the same as we had previously reported (Jafarzadeh et al., 2014c ▶). Briefly, the ginger-treated EAE mice exhibited lower clinical score of EAE, a delay in disease onset and lower infiltration of the inflammatory cell into the spinal cord. Moreover, loss of the body weight was lesser in 300 mg/kg ginger-treated EAE group. 

The presence of EAE was approved according to the clinical signs of disease and histopathological observations of the CNS (Takeuchi C et al., 2013 ▶; Robinson et al., 2014 ▶). The PBS-treated EAE mice showed the clinical symptoms of EAE on day 10, whereas 200 and 300 mg/kg ginger-treated EAE groups exhibited the disease signs on days 14 and 15, respectively. The maximum mean clinical score (MMCS) for PBS-treated EAE group was 5.00 ± 00 whereas this parameter was 2.40 ± 0.24 and 2.20 ± 0.20 for 200 and 300 mg/kg ginger-treated EAE groups, respectively. The MMCS in 200 and 300 mg/kg ginger-treated groups was significantly lower as compared to PBS-treated EAE mice (p<0.002 and p<0.001, respectively). No significant difference was observed between 200 and 300 mg/kg ginger-treated EAE groups regarding the MMCS.

**The effect of ginger extract on gene expression of IL-12 in the spinal cords**

**Figure 1:**
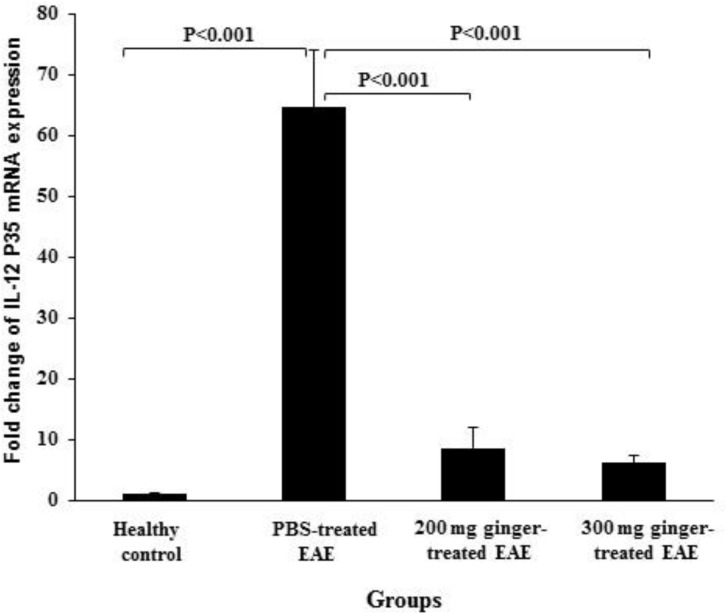
Comparison of the expression of IL-12 P35 mRNA between ginger-treated and control groups. Gene expression of IL-12 P35 in PBS-treated EAE group was significantly higher than that in healthy group. In both 200 and 300 mg/kg ginger-treated EAE groups, the expression of IL-12 P35 mRNA was significantly lower than that in PBS-treated EAE group

**Figure 2:**
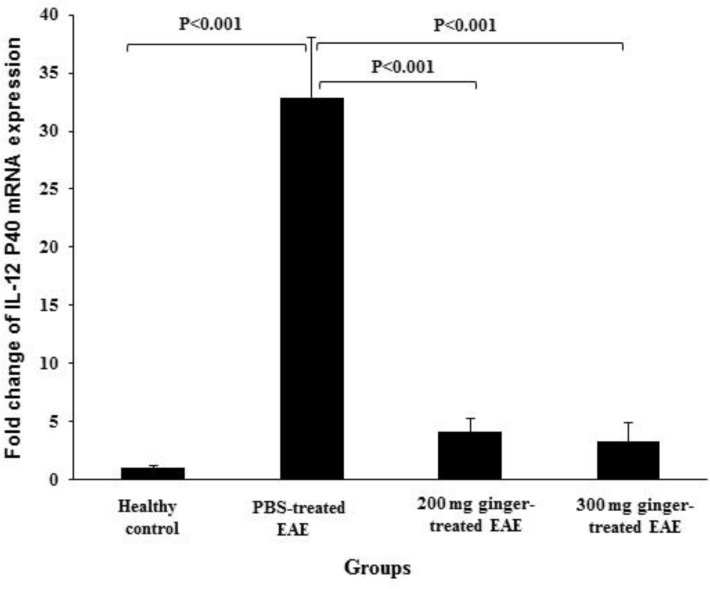
Comparison of the expression of IL-12 P40 mRNA between ginger-treated and control groups. Gene expression of IL-12 P40 in PBS-treated EAE group was significantly higher than that in healthy group. The expression of IL-12 P40 mRNA in 200 and 300 mg/kg ginger-treated EAE mice was significantly lower than that in PBS-treated EAE group

The expression of IL-12 P35 and IL-12 P40 mRNA were 0.98 ± 0.16 and 0.96 ± 0.26 in healthy control group, 64.58 ± 9.36 and 32.87 ± 5.13 in PBS-treated EAE mice, 8.55 ± 3.40 and 4.10 ± 1.12 in 200 mg/kg ginger-treated EAE mice, 6.05 ± 1.15 and 3.23 ± 1.65 in 300 mg/kg ginger-treated EAE group, respectively (Figures 1and 2).

In PBS-treated EAE mice, the expression of IL-12 P35 and IL-12 P40 mRNA were significantly higher than in healthy normal group (p<0.001). In both 200 and 300 mg/kg ginger-treated EAE groups, the expression of IL-12 P35 and IL-12 P40 mRNA was significantly lower as compared to PBS-treated EAE mice (p<0.001). 

The expression of IL-12 P35 mRNA in both 200 and 300 mg/kg ginger-treated EAE groups was significantly higher as compared to healthy control group (p<0.05 and p<0.01, respectively). No significant difference was observed between 300 mg/kg ginger-treated EAE mice and healthy control group regarding the expression of IL-12 P40 mRNA. However, the expression of IL-12 P40 mRNA in 200 mg/kg ginger-treated EAE group was significantly higher as compared to healthy control group (p<0.02). 

**The effect of ginger extract on gene expression of TGF-β in the spinal cords**

**Figure 3:**
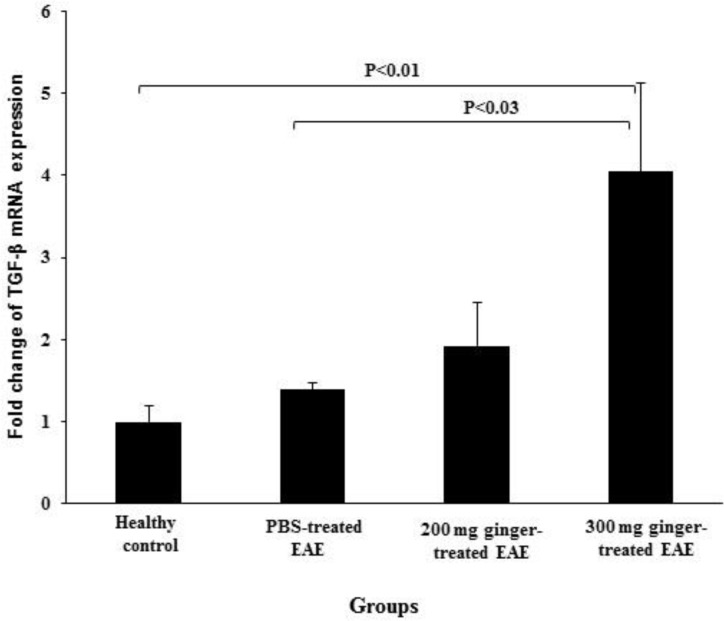
Comparison of the expression of TGF-β mRNA between ginger-treated and control groups. No significant difference was observed between PBS-treated EAE mice and healthy group regarding gene expression of the TGF-β. The expression of TGF-β in 300 mg/kg ginger-treated EAE group was significantly higher than that in healthy control group and PBS-treated EAE mice. No significant difference was observed between 200 mg/kg ginger-treated EAE group and PBS-treated EAE mice or healthy control group regarding gene expression of the TGF-β

The expression of TGF-β mRNA was 1.00 ± 0.19 in healthy control group, 1.39 ± 0.08 in PBS-treated EAE mice, 1.92 ± 0.54 in 200 mg/kg ginger-treated EAE mice and 4.05 ± 1.07 in 300 mg/kg ginger-treated EAE group (Figure 3). No significant difference was observed between PBS-treated EAE mice and healthy control group regarding gene expression of the TGF-β. In 300 mg/kg ginger-treated EAE group, the expression of TGF-β mRNA was significantly higher as compared to healthy control group and PBS-treated EAE mice (p<0.01 and p<0.03, respectively). No significant difference was observed between 200 and 300 mg/kg ginger-treated EAE groups regarding the expression of TGF-β mRNA, although this parameter was found to be higher in 300 mg/kg ginger-treated EAE mice.

**The effect of ginger extract on serum levels of IL-12**

**Figure 4:**
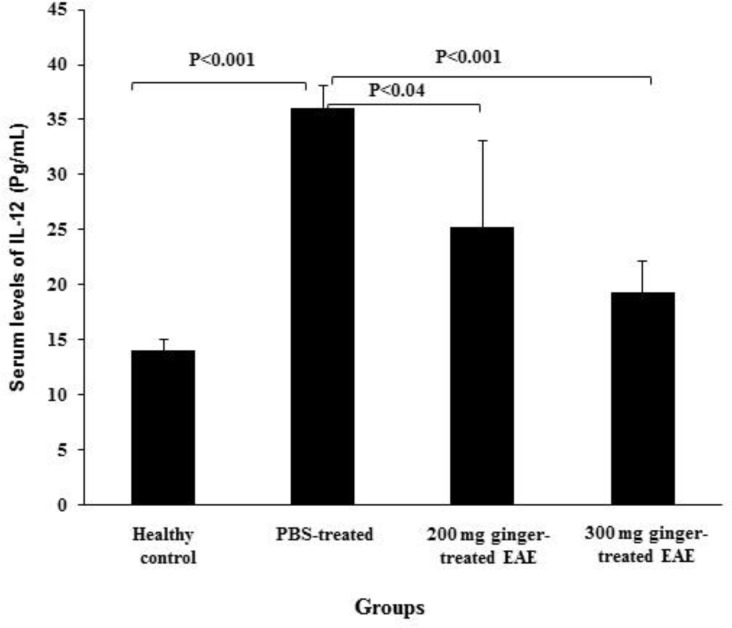
Comparison of serum levels of IL-12 between ginger-treated and control groups. Serum IL-12 levels in PBS-treated EAE mice were significantly higher than those in healthy control group. The levels of IL-12 in 200 and 300 mg/kg ginger-treated EAE groups were significantly lower than those in PBS-treated EAE mice

The mean serum levels of IL-12 were14.02 ± 1.05 pg/ml in healthy control group, 36.00 ± 2.14 pg/ml in PBS-treated EAE mice, 25.25 ± 7.88 pg/ml in 200 mg/kg ginger-treated EAE mice and 19.30 ± 2.90 pg/ml in 300 mg/kg ginger-treated EAE group (Figure 4). The mean serum levels of IL-12 in PBS-treated EAE mice was significantly higher as compared to healthy control group (p<0.001). The mean serum levels of IL-12 in 200 and 300 mg/kg ginger-treated EAE groups were significantly lower as compared to PBS-treated EAE mice (p<0.04 and p<0.001, respectively). No significant difference was observed between 200 and 300 mg/kg ginger-treated EAE groups regarding serum IL-12 levels or its expression in the CNS.

**The effect of ginger extract on serum levels of TGF-β**

**Figure 5:**
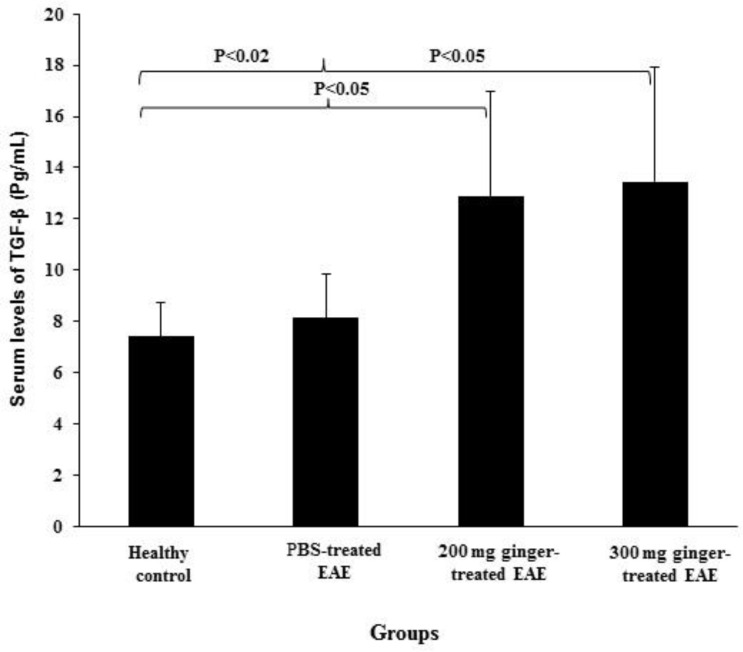
Comparison of serum levels of TGF-β between ginger-treated and control groups. No significant difference was observed between PBS-treated EAE mice and healthy control group regarding serum TGF-β levels. Serum TGF-β levels in 200 mg/kg ginger-treated EAE group were significantly higher than those in healthy control group and PBS-treated EAE mice. In 300 mg/kg ginger-treated EAE group, serum TGF-β levels were also significantly higher as compared to healthy control group and PBS-treated EAE mice

The mean serum levels of TGF-β were7.40 ± 1.35 pg/ml in healthy control group, 8.13 ± 1.69 pg/ml in PBS-treated EAE mice, 12.89 ± 4.10 pg/ml in 200 mg/kg ginger-treated EAE mice and 13.45 ± 4.48 pg/ml in 300 mg/kg ginger-treated EAE group (Figure 5). In 200 mg/kg ginger-treated EAE group, the mean serum levels TGF-β were significantly higher as compared to healthy control group and PBS-treated EAE mice (p<0.05). In 300 mg/kg ginger-treated EAE group, the mean serum levels of TGF-β were also significantly higher as compared to healthy control group and PBS-treated EAE mice (p<0.02 and p<0.05, respectively)

## Discussion

We recently demonstrated that treatment of EAE mice with ginger extract suppresses the development of EAE. The clinical symptoms of EAE were appeared later and the clinical scores of disease were also lower in ginger-treated EAE mice as compared to PBS-treated EAE (Jafarzadeh et al., 2014c ▶). The results of the present study demonstrated that gene expression of IL-12 in the spinal cords and its serum levels were significantly higher in PBS-treated EAE group as compared to normal control mice. 

IL-12 may play a major role in EAE development through induction of differentiation of both Th1 and Th17 cells (Lasek et al., 2014 ▶). It has been reported that both Th1 and Th17 cells may play a complementary role in the pathogenesis of EAE disease (Fletcher et al., 2010 ▶). Indeed, mice with defect in either RORγt (a Th17-specific transcription factor) or T-bet (a Th1-specific transcription factor) were resistant to EAE induction (Fletcher et al., 2010 ▶). Moreover, both Th1- and Th17 cells are accumulated in the CNS during EAE development (Fletcher et al., 2010 ▶; Raphael et al., 2015 ▶). Th1 cells induce macrophage-rich infiltrates into the spinal cord whereas Th17 cells promote neutrophils aggregation, especially in the brain (Kroenke et al., 2008 ▶; Fletcher et al., 2010 ▶). Moreover, IFN-γ (a main cytokine of Th1 cells) may play a key role in the pathogenesis of EAE disease thorough promoting M1 macrophages expansion (Dungan et al., 2014 ▶). Furthermore, IFN-γ up-regulates the inducible nitric oxide synthase (NOS) resulting in high levels of NO production by DC and macrophages. Therefore, it has been reported that the IL-12/IFN-γ/NO axis plays a critical role in the development of EAE (Xiao et al., 2008 ▶). 

It has been also reported that IL-7 is essential for expansion of Th1 and Th17 cells in EAE and MS disease (Liu et al., 2010 ▶; Lee et al., 2011 ▶). It has been also demonstrated that IL-12 induces the expression of IL-7 in microglia, macrophages and astrocytes (Jana et al., 2014 ▶). Accordingly, IL-12/IL-7 axis may also participate in the development of the EAE through reinforcing both Th1- and Th17 cells-related responses. Based on the mentioned explanations, our observations confirm that IL-12 may play a critical role in the pathogenesis of EAE. 

Our results also demonstrated that treatment of EAE mice with ginger extract decreases the expression of IL-12 either in the CNS or serum. In both 200 and 300 mg/kg ginger-treated EAE groups, the expression of IL-12 was lower as compared to PBS-treated EAE mice. No significant differences were observed among ginger-treated EAE groups and normal group regarding the expression of IL-12. In agreement with our findings, it has been reported that ginger extracts suppress IL-12 secretion by LPS-stimulated macrophage (Tripathi et al., 2008 ▶). The exact mechanisms through which ginger extract may influence the IL-12 production remain to understand in future studies. However, ginger extract may directly and/or indirectly modulate IL-12 production. 

It should be noted that IL-12 –induced Th1 cells produce IFN-γ which initiates a positive feedback loop by more activating macrophages and result in more production of IL-12. In our previous study, lower serum levels of IFN-γ were observed in ginger-treated EAE mice as compared to PBS-treated EAE group (Jafarzadeh et al., 2014c ▶). Therefore, ginger extract may also exert an inhibitory effect on the production of IL-12 indirectly through suppressing IFN-γ synthesis. 

It has been reported that the production of IL-12 during the immune response can be increased through toll-like receptors ligation (such as TLR4 ligation with lipopolysaccharide), signalling through cytokines (such as IL-1β) or direct cell–cell communication (such as CD40L–CD40 interaction). Suppression of IL-12 production is also mediated by cytokines such as type I IFNs, IL-10 and TGF-β as well as prostaglandin E2 (PGE2), suppressive molecules [such as T-cell immunoglobulin and mucin domain-containing protein 3 (Tim-3)], CTLA-4 and CD200] (Lasek et al., 2014 ▶). Accordingly, ginger extract may decrease IL-12 expression through inhibitory effects on the IL-12 inducing factors (including TLRs, IL-1β and CD40L–CD40) and/or through stimulatory effects on the IL-12 suppressor factors (including type I IFNs, IL-10, TGF-β, Tim-3, CTLA-4 and CD200). It has been also demonstrated that TGF-β has inhibitory effects on IL-12 production by monocytes (Mantel et al., 2011 ▶). The results of the present study also demonstrated that the ginger extract significantly increased the expression of TGF-β that may account for lower expression of IL-12 in ginger-treated EAE mice. Accordingly, the decreasing effects of ginger extract on IL-12 expression may modulate both Th1 and Th17 responses and eventually lead to amelioration of EAE. 

The results of the present study also indicated that there are no significant differences between PBS-treated EAE mice and healthy control group regarding the expression of TGF-β. Although, some defects have been reported in the number or function of Treg cells (the main producers of TGF-β) from MS patients and EAE models (Buc, 2013c ▶; Lowther and Hafler, 2012); however, the results of our recent study showed that there was no significant difference between MS patients and healthy control group regarding serum levels of a Treg cells-related cytokine IL-35 (Jafarzadeh et al., 2014d ▶). Recently, it has been reported that serum levels of TGF-β were decreased in the development stage of EAE (Lu et al., 2014 ▶). In the present study, we measured cytokines expression on day 31 post immunization. Accordingly, diminished levels of TGF-β may contribute to EAE development during early phase of disease.

It should be noted that the presence of the balance between Th17- and Treg cells-related responses is crucial for immune homeostasis. An imbalance in Th17/Treg cells-related responses with tendency toward Th17 cells response may contribute to the development of EAE and probably human MS. Indeed, higher frequencies of auto-reactive Th17 cells were demonstrated in the CNS of MS patients and in EAE mice (Raphael et al., 2015 ▶). TGF-β is required for both Th17- and Treg cells differentiation. Stimulation of naive T cells in the presence of TGF-β leads to the Treg cells differentiation, whereas a combination of TGF-β and IL-6 results in TH17 cell differentiation (Zhang et al., 2014 ▶). It has been demonstrated that the expression of IL-6 mRNA is significantly increased in the brain during development of EAE (Murphy et al., 2010 ▶). It has been also reported that when anti-IL-6R antibodies were injected immediately after MOG immunization, development of EAE was inhibited and no Th17 cells were found in the draining lymph nodes or the spinal cord (Kimura et al., 2010 ▶). 

It seems that TGF-β may have a pathologic or protective role during the EAE in the presence of local high or low IL-6 concentration, respectively. The presence of higher levels of IL-6 during initial stage of EAE development leads to the differentiation of naïve Th cells into pathogenic Th17 cells in the presence of adequate levels of TGF-β. Accordingly, at initial stage of the development of EAE, TGF-β may serve as a Th17 cells inducing factor due to the presence of high levels of IL-6. When EAE is developed, TGF-β may have an enhancing effect on EAE severity due to the presence of other cytokines (especially IL-6) and various inflammatory cells in CNS. It has been also reported that TGF-β can induce both Th17 and Th9 responses depending on the existence of other cytokines and cellular types (Zheng, 2013 ▶). In addition to Th17 cells, the participation of Th9 cells has been also reported in the pathogenesis of MS and EAE diseases (Raphael et al., 2015 ▶). 

The results of the present study also demonstrated that ginger extract at a dose of 300 mg/kg significantly increased the expression of TGF-β. On the other hand, the inhibitory effects of ginger and its derivative on the IL-6 production have been demonstrated in other studies (Lee et al., 2012 ▶; Ho et al., 2013 ▶). Accordingly, treatment with ginger extract may improve the Th17/Treg cells imbalance with tendency toward Treg cells differentiation. Therefore, higher levels of TGF-β and lower levels of IL-6, TGF-β lead to the differentiation of protective Treg cells that may play an important role in the amelioration of EAE. 

It should be noted that other pro-inflammatory cytokines and chemokines such as IL-6, IL-12, IL-23 and GM-CSF may also play important roles in the regulation of EAE development (Liu et al., 2014 ▶). Therefore, evaluation the effects of ginger extract on the expression of other immunological parameters is important for understanding possible molecular mechanisms of ginger extract during EAE development. 

In conclusion, our results showed higher expression of IL-12 in the spinal cord and serum of EAE mice. Accordingly, the up-regulation of the expression of IL-12 may be involved in the development EAE. Moreover, treatment of EAE mice with ginger extract modulates the expression of IL-12 and TGF-β in CNS and serum of mice with EAE. Further studies should be conducted to evaluate the possible therapeutic potential of ginger extract or its derivative in the treatment of EAE or MS diseases.
